# Quality of Life of Patients With Hidradenitis Suppurativa in Jeddah, Saudi Arabia

**DOI:** 10.7759/cureus.20234

**Published:** 2021-12-07

**Authors:** Awadh M Alamri, Abeer A Alzahrani, Anan M Aldakhil, Heba E Alharbi, Farah A Yahya

**Affiliations:** 1 Dermatology, King Abdualziz Medical City, Ministry of National Guards - Health Affairs, Jeddah, SAU; 2 College of Medicine, King Saud Bin Abdulaziz University for Health Sciences, Jeddah, SAU

**Keywords:** acne, acne inversa, heath related quality of life, hidradenitis suppurativa, patient-reported outcome

## Abstract

Background* *

Hidradenitis suppurativa (HS) is a chronic inflammatory skin condition that affects the apocrine gland-bearing areas of the body. It initially presents as painful nodules that eventually develop into abscesses, draining sinuses, and scarring. These manifestations have physical and psychological impacts, which lead to poor quality of life. This study examined the association between quality of life and disease severity, as well as identified the areas of the body most affected by HS among patients in Saudi Arabia.

Methods

This cross-sectional study examined patients with HS who were seen at two dermatology outpatient clinics between December 2018 and March 2019. The patients completed a self-administered standardized questionnaire on the Dermatology Life Quality Index (DLQI).

Results

The average DLQI score was 15.39 ± 8.37. The majority of patients were classified as stage 3, which indicated that HS has a very large effect on quality of life. The right and left axillae were the most commonly affected areas of the body, with 80.6% of patients noting involvement. While the mean DLQI score was higher in males compared to females, there was no significant difference between the two groups (16.44 ± 9.01 vs. 13.08 ± 6.65; *P* = 0.248).

Conclusion

HS caused significant impairment in the quality of life of patients with HS in Saudi Arabia. The mean DLQI score in our study was higher than the score previously reported in the literature. Further studies may identify opportunities to provide additional awareness, care, and support for patients with HS in Saudi Arabia.

## Introduction

Hidradenitis suppurativa (HS) is a chronic inflammatory condition of the apocrine glands that is characterized by hair follicle inflammation, abscess formation, and scarring [1]. HS most commonly affects the axillae, breasts, and genital area [2].The physical symptoms of HS may prompt feelings of embarrassment, self-consciousness, isolation, and depression, which interfere with quality of life [3,4]. Studies in Greece, Poland, Denmark, and Canada demonstrated that HS significantly influenced the quality of life [3-6]. Assessment of the quality of life is essential to determine the impact of the disease on the patients. However, there are no research papers conducted to estimate the impact of HS on the patients’ quality of life in the middle eastern region. Therefore, this study helps in understanding the impact of HS on the patients more. Finally, the results of this study help to improve the health care for HS patients by determining their specific needs for further educational and psychological support, self-management, treatment modalities, and multidisciplinary care. 

## Materials and methods

This cross-sectional, multicenter study was conducted in the dermatology department’s outpatient clinics of two tertiary hospitals which are King Fahad General Hospital and King Abdulaziz Medical City, Jeddah, Saudi Arabia. This study was approved by the institutional review board (IRB) at King Abdullah International Medical Research Center (KAIMRC) with an approval number RYD-18-417780-141769. Consecutive sampling technique was used, and the patients diagnosed with HS who consulted between December 2018 and March 2019 were included in this study. The patients who participated in the study were all diagnosed with HS clinically and the diagnosis was documented in their medical files. We excluded patients with other skin diseases, such as folliculitis, furunculosis, psoriasis, vitiligo, severe eczema, alopecia, and skin cancers. All patients with mental or psychological illnesses were excluded. All the patients who participated in the study have signed a consent form.

Patients completed a previously validated self-administered standardized questionnaire on the Dermatology Life Quality Index (DLQI) [7]. The questionnaire is composed of three parts and includes sections on demographic data, disease characteristics (with an illustration of a human body to document the affected regions) and the Hurley staging classification [8], and the DLQI. The DLQI scores were interpreted as follows: 0-1, no effect at all on the patient's life; 2-5, small effect on the patient's life; 6-10, moderate effect on the patient's life; 11-20, very large effect on the patient's life; and 21-30, extremely large effect on the patient's life. The daily clinic nurses in both outpatient clinics were trained to deliver this survey.

The data were collected and analyzed using SPSS for Windows version 21.0 (IBM, Armonk, New York, USA). The demographic characteristics of the patients were presented with descriptive statistics, such as mean, standard deviation, frequencies, and percentages. Associations between variables were tested with the Student’s t-test, analysis of variance test, and chi-square test, as necessary. We examined whether there was a significant difference in the quality of life among patients with different severities of HS. We further analyzed whether there was an association between disease severity and quality of life.

## Results

**Figure 1:**
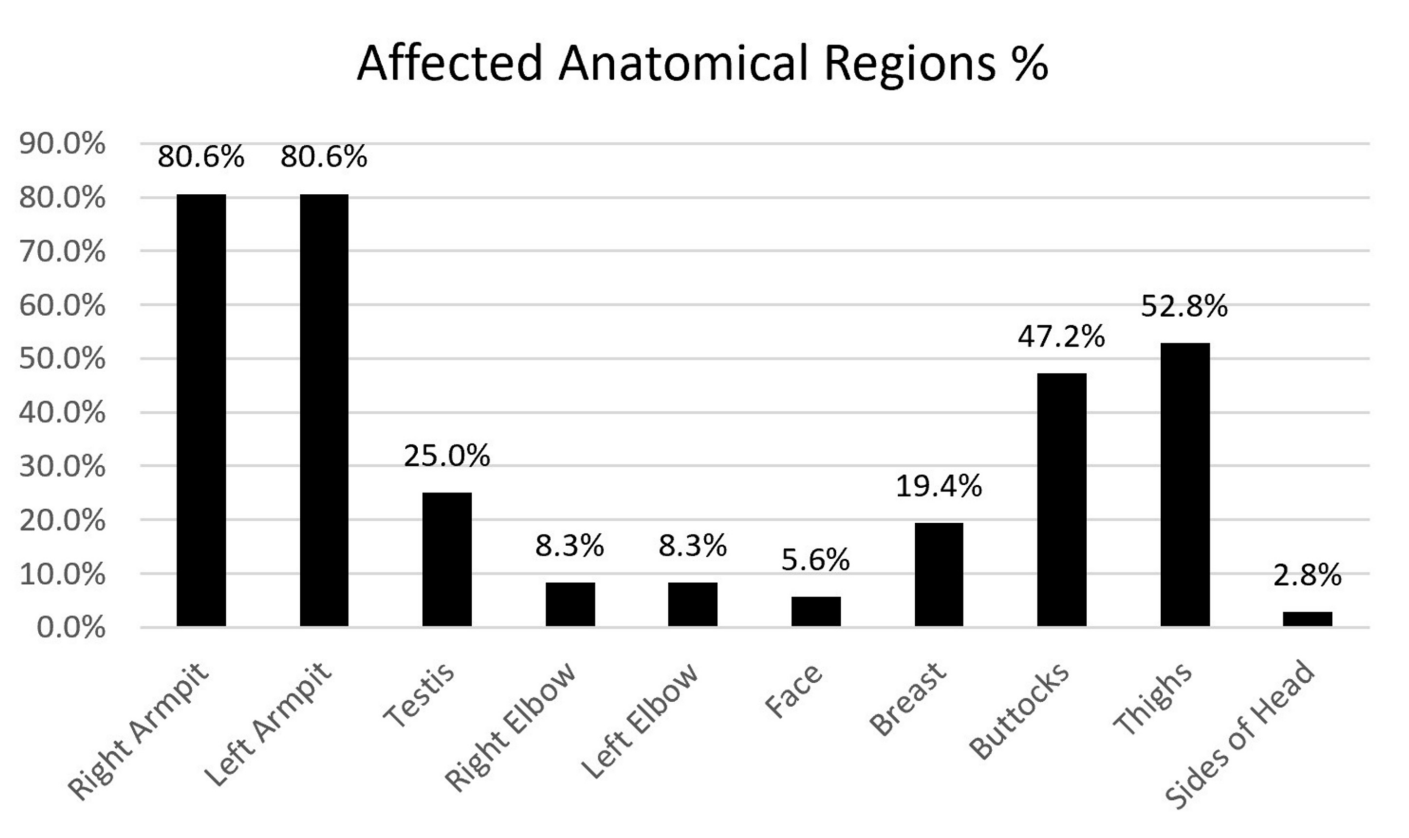
Multiple response analysis of affected anatomical regions Involved sites in our study population

A total of 36 patients were included in this study. Twenty-four (66.7%) patients were men, and 12 (33.3%) patients were women. The mean age of the participants was 35.9 ± 10.7 years (range, 18-65). The mean Hurley staging score was 16.1 ± 8.7, which classified most patients with stage 3 HS. The average DLQI score was 15.3 ± 8.3, which indicated that HS has a “very large effect on the patient’s life” (Table 1). The right and left axillae were the most commonly affected areas; right and left axillary involvement was each noted in 80.6% of all respondents (Figure 1).

**Table 1:** Demographic and clinical characteristics of the study population Data were presented as mean ± standard deviation or numbers with percentages.

Variables	Patients (N = 36)
Age in years	35.9 ± 10.7
Gender	
Male	24 (66.7)
Female	12 (33.3)
Hurley Stages	
Stage 1	7 (21.1)
Stage 2	9 (27.3)
Stage 3	17 (51.5)
Dermatology Life Quality Index score	15.3 ± 8.3

The mean DLQI score in male and female patients was 16.4 ± 9.01 and 13.08 ± 6.6, respectively; however, there was no significant difference between these two groups (*P* = 0.248). There was also no significant association between age and disease burden (*P* = 0.580). There was no significant difference in DLQI scores among the Hurley stages and no significant association between the Hurley stage and DLQI score (*P* = 0.416) (Tables 2, 3). Three participants were unstagable; thus, they were not included in the Hurley staging and DLQI comparison.

**Table 2:** Comparison between the Hurley stage and Dermatology Life Quality Index (DLQI) scores

Hurley Stage	DLQI Score	*P-*value
Stage 1	11.1 ± 7.3	0.416
Stage 2	15.3 ± 8.2	
Stage 3	16.1 ± 8.7	

**Table 3:** Association between the Hurley stage and Dermatology Life Quality Index (DLQI) scores

DLQI Score Interpretation	Hurley Stage		
Effect on the Quality of Life	Stage 1	Stage 2	Stage 3
No to small effect	2 (33.3%)	1 (16.7%)	3 (50%)
Moderate to very large effect	4 (23.5%)	5 (29.4%)	8 (47.1%)
Extremely large effect	1 (10%)	3 (30%)	6 (60%)

## Discussion

HS is a chronic inflammatory skin disease of the pilosebaceous unit. It affects intertriginous areas of the body such as the axillae, buttocks, groins, and submammary folds [9]. Tzellos et al. demonstrated that HS impacts the physical and psychological aspects of quality of life [3,10,11]. HS begins with blockage of the hair follicle, which promotes inflammation, dysregulation of the immune system, and eventual bacterial infection. Infected hair follicles are likely to rupture, which releases bacteria and keratin into the dermis layer of the skin. This activates immune cells in the dermis, resulting in abscess formation [2].

Our data correlated well with previous literature on the chronicity and impact of HS. Among patients with HS in Saudi Arabia, HS had a very high impact on the quality of life at all stages of the disease. The mean DLQI score in our study population was 15.39 ± 8.37, which indicated that the disease had a great impact on the quality of life regardless of the Hurley stage. The mean DLQI in our study was comparable to those reported by Matusiak et al. 12.7 ± 7.7, Frings et al. 12 ± 7.0, Jorgensen et al. 11.9 ± 7.6, Kourins et al. 11.43 ± 6.61, and Schneider-Burrus et al. 13.18 ± 0.37 [4,7,12-14].

While previous studies showed a female predominance, HS preferentially affected males in our study population. However, this observation could be limited by the small sample size included in the study. In our study, there was no association between disease severity and age. Similarly, Schneider-Burrus et al. demonstrated no actual relation between the patient’s age and the impact of HS on the quality of life [14].

The most burdensome characteristics of HS are the pain that is experienced by 97% of the patients diagnosed with HS. The patients report that it limits their daily physical activities including household chores and exercising. As a result of the recurrent painful abscesses, the lesions may result in malodorous discharge which the patients are conscious of, and they often fear others' reactions. Additionally, the patients might feel embarrassed about visible active lesions or scars, and HS may, therefore, lead to stigma [15]. HS can affect all aspects of the patient’s life and it has been associated with feelings of distress, depression, and other psychological symptoms. The physical symptoms of HS also lead to feelings of shame and social isolation [5,16]. The decrease in quality of life might be different depending on the affected anatomical region. HS with genital localization was associated with the strongest negative impact on quality of life and causes great disability affecting the patient’s intimate relationships and sexual function. A study conducted in 13 European countries reported that sexual dysfunction was higher in HS patients compared to various other dermatological diseases [7,17]. However, our study demonstrated that the most affected anatomical regions by HS were the right and left armpits both of which had a percentage of 80.6%. 

Moreover, the nature of the disease and its recurrent flares can have a great impact on work as well leading to absent days and increased burden. Matusiak conducted a survey, which demonstrated that approximately 58% of patients with HS lost 34 days of work annually due to HS [7]. A Polish study done by Kaaz et al. showed that HS is also associated with a poorer quality of sleep compared to control [18].

## Conclusions

In conclusion, HS is a serious chronic skin condition with physical and psychological implications. It clearly causes a significant impairment in quality of life among patients in Saudi Arabia. HS is a rare condition, and the small sample size of our study, despite data being collected from two centers, was a significant limitation of this study. We recommend conducting further multicenter studies with larger sample sizes. Future studies should examine the factors that could provide additional awareness, support, and care for patients with HS.

## Figures and Tables

**Figure 2:**
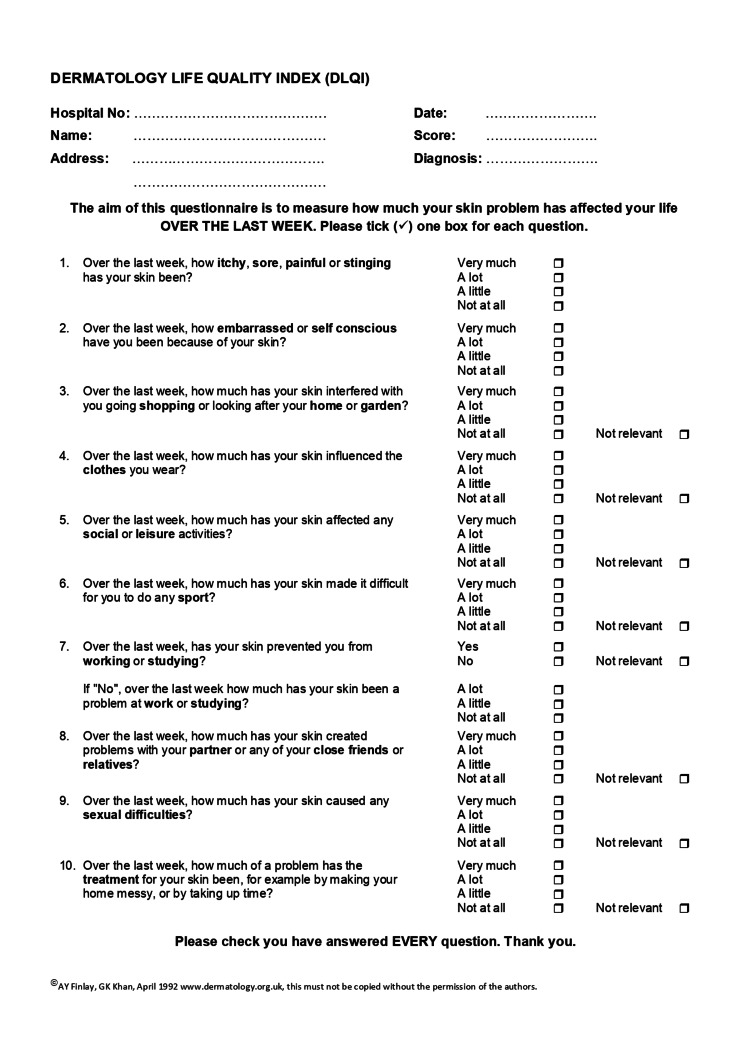
Dermatology Life Quality Index (DLQI)

**Figure 3:**
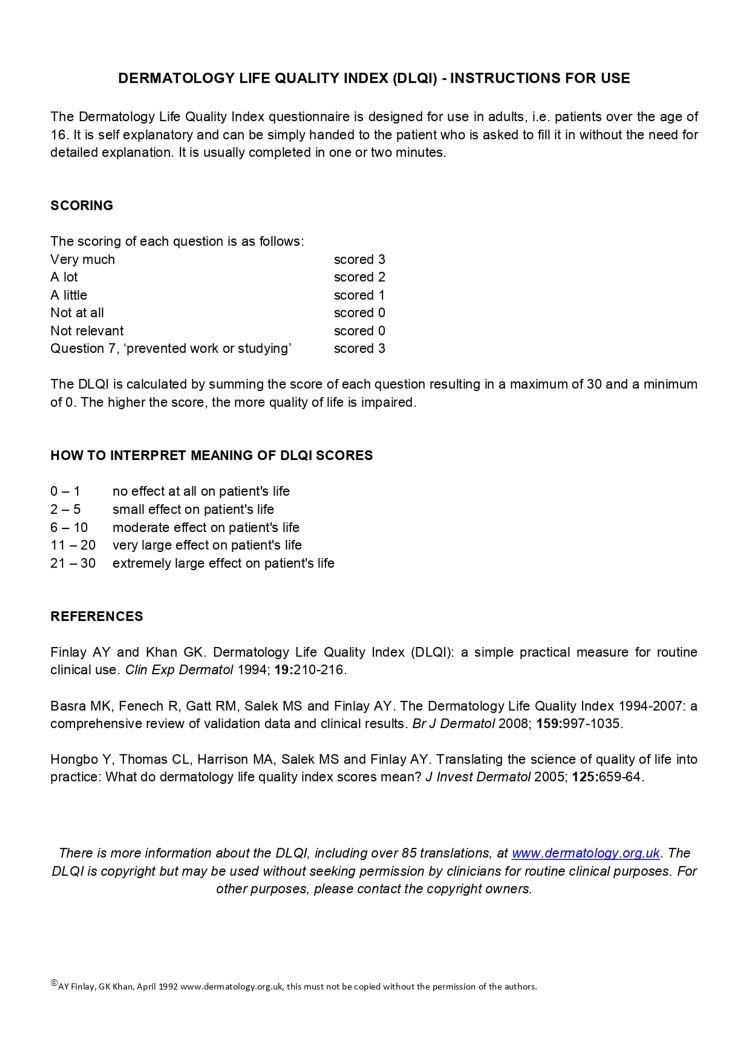
Dermatology Life Quality Index (DLQI) (Continued)

## Appendices

Dermatology Life Quality Index (DLQI) 
