# Correction: Quality of Life of Patients With Hidradenitis Suppurativa in Jeddah, Saudi Arabia

**DOI:** 10.7759/cureus.c394

**Published:** 2026-01-09

**Authors:** Awadh M Alamri, Abeer A Alzahrani, Anan M Aldakhil, Heba E Alharbi, Farah A Yahya

**Affiliations:** 1 Dermatology, King Abdualziz Medical City, Ministry of National Guards - Health Affairs, Jeddah, SAU; 2 College of Medicine, King Saud Bin Abdulaziz University for Health Sciences, Jeddah, SAU

This article has been updated to add an additional affiliation for Awadh Alamri.

